# Case report of simultaneous *phlegmasia cerulea dolens* and acute limb ischemia

**DOI:** 10.1016/j.ijscr.2024.110596

**Published:** 2024-11-12

**Authors:** Smoter Šimon, Slyško Roman

**Affiliations:** Department of Vascular surgery, University hospital of Bratislava, Faculty of Medicine – Comenius University, Slovak Republic

**Keywords:** *Phlegmasia cerulea dolens*, *Phlegmasia alba dolens*, Acute limb ischemia, Embolectomy, Catheter-directed thrombolysis, Pelvic tumour, Case report

## Abstract

**Introduction:**

*Phlegmasia cerulea dolens* (PCD) is a rare, life- and limb-threatening condition of acute massive deep vein thrombosis (DVT) that requires emergent therapeutic intervention. While endovascular therapy is the preferred treatment approach, we describe a case where surgical embolectomy is an alternative approach in specific scenarios. In our case, we presented a patient with simultaneous PCD and acute limb ischemia (ALI).

**Case report:**

A patient presented with haematuria, abdominal pain, cyanotic changes in the limb, and sensory/motor deficits in the right lower limb. Imaging tests revealed thromboembolic occlusion of both the arterial and venous systems and a bleeding tumour in the pelvic region. Due to the rapid progression of symptoms, macroscopic haematuria with anaemia, renal failure, and the necessity for intervention in both the arterial and venous systems, we performed a successful simultaneous surgical embolectomy of the arterial and venous systems right lower limb, resulting in prompt symptom resolution.

**Discussion:**

*Phlegmasia cerulea dolens* is a rare manifestation of massive iliofemoral deep vein thrombosis. Its pathophysiology can indirectly compromise arterial patency. Treatment options include endovascular therapy - catheter-directed thrombolysis (CDT), percutaneous mechanical thrombectomy (PMT) or surgical intervention. These options carry risks, including damage to the venous endothelium or periprocedural pulmonary embolism. This case report demonstrates a situation where surgical intervention was a viable option. We presented a more endothelium-sparing surgical approach using a sterile compression bandage during embolectomy.

**In conclusion:**

To date, there are no established guidelines from professional societies regarding the treatment of PCD. The authors emphasize the need for an individualized approach to patients with PCD. They also highlight the importance of access to comprehensive treatment, including both endovascular and surgical methods.

**Methods:**

work has been reported in line with SCARE criteria.

## Highlights

• Phlegmasia cerulea dolens (PCD) is a rare and serious condition caused by a massive blood clot in the deep veins of the leg.
• Treatment options include endovascular therapy or surgical intervention.
• Endovascular therapy is preferred, but surgery may be necessary in some cases.
• This case highlights the importance of surgeons being skilled in both endovascular and surgical techniques.

## 1 Introduction

*Phlegmasia cerulea dolens* (PCD) is a rare form of deep vein thrombosis characterized by acute swelling, ischemic pain, and cyanosis of the limb [1]. The term phlegmasia was first described by Fabricius Hildanius in the 16th century. In 1938, it was further classified into two categories: *phlegmasia cerulea dolens*, characterized by painful blue inflammation and *phlegmasia alba dolens*, also known as “milk leg,” which refers to painful white inflammation [2]. PCD is a progression of phlegmasia alba dolens, characterized by subtotal occlusion of both major and collateral venous channels. However, unlike venous gangrene, it can be reversible. Venous hypertension due to subtotal or complete occlusion disrupts the balance between hydrostatic and oncotic pressures. The increase in compartment pressure leads to compression of the arterial system, potentially causing acute limb ischemia, and also to fluid leakage into the interstitial space, worsening limb oedema and potentially leading to hypovolemic shock [3]. The final stage of massive thromboembolism is irreversible venous gangrene, with a 50 % risk of limb amputation and a 40 % mortality rate [4]. Diagnosis relies primarily on physical examination, patient history, and clinical findings. Duplex sonography is used to verify venous thrombosis. The initial step of medical management involves immediate limb elevation to reduce oedema and pressure on the arterial system. Medical management includes anticoagulation therapy and fluid resuscitation [5]. The mainstay of medical treatment is unfractionated heparin at a dose of 10–15 units per kilogram, followed by maintaining an activated partial thromboplastin time (aPTT) 1.5 to 2 times the reference value. Endovascular or surgical intervention represents the definitive treatment approach. Several studies have confirmed the clinical effectiveness of CDT. Percutaneous mechanical thrombectomy (PMT) can be an effective alternative or complementary therapy, with some studies suggesting its advantage over CDT due to lower thrombolytic infusion time [6]. All endovascular procedures carry a risk of pulmonary embolism due to embolus fragmentation and instrumentation manipulation. Despite a mortality rate of pulmonary embolism, vena cava filter placement is not routinely performed. Caval filter placement is the only alternative for patients with contraindications to anticoagulation therapy. Furthermore, there are documented cases where filters have become a nidus for thrombus formation, potentially exacerbating PCD [7]. Surgical treatment also has a role in therapy. Restoration of venous outflow can be achieved through catheter thrombectomy, gradually loaded compression bandages, or a combination of these methods [8]. Despite early endovascular or surgical intervention, the risk of developing post-thrombotic syndrome and recurrence remains high [2].

## 2 Case report

**Fig. 1:**
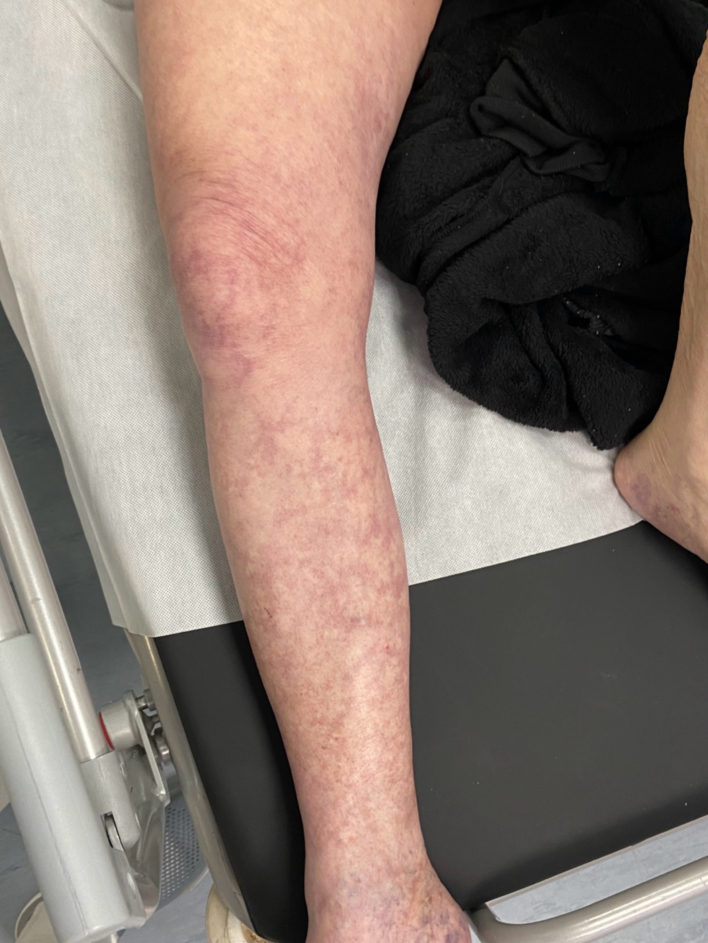
Clinical presentation at emergency department.

**Fig. 2:**
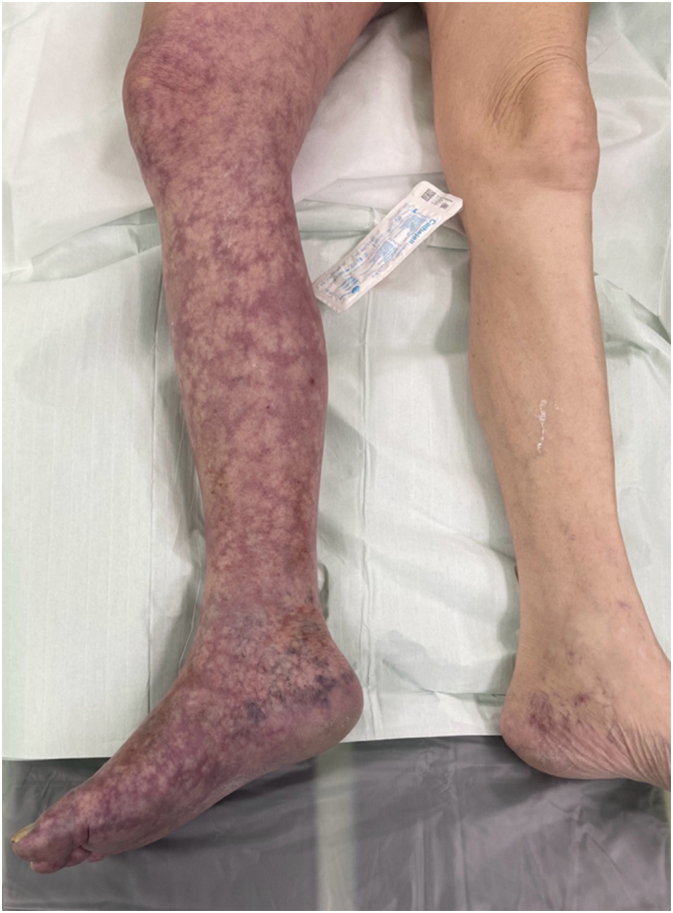
Evolution of clinical findings within 45 min.

A 73-year-old male smoker, without prior medical history or medication use, presented to the emergency department with acute, progressively worsening pain in his right lower extremity over approximately two hours. He reported numbness in the sole of his right foot and an inability to move his toes and ankle. The patient reported macrohematuria for about two weeks but denied any prior walking difficulties, claudication, rest pain, or swelling. Physical examination of the right lower limb revealed absent pulses in the femoral, popliteal, and distal arteries. The limb was cool from the toes to mid-calf and displayed a pale skin colour. (Fig. 1, Fig. 2) Sensory and motor deficits were also noted. Abdominal examination revealed significant meteorism, generalized tenderness, and bilateral flank pain upon palpation. Laboratory tests showed signs of acute renal failure (eGF-CKD-EPI 0.26 ml/s, creatinine level 320 μmol/l, urine output was 0.4 mL/kg/h) and anaemia (haemoglobin level 84 g/l). The patient's vital signs were stable upon arrival at the emergency department: systolic blood pressure 105 mmHg, diastolic blood pressure 60 mmHg, pulse rate 77 beats per minute, and oxygen saturation 94 %. Vital signs remained stable throughout the surgical procedure and the postoperative period.

**Fig. 3:**
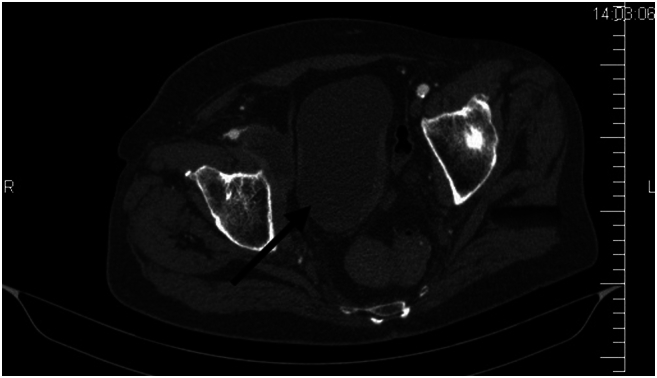
CT scan revealing a neoplastic mass within the pelvic cavity. The arrow points to the mass, which is compressing the right iliac vein.

An acoustic Doppler examination confirmed a triphasic flow in the common femoral artery with peak systolic velocity (PSV) up to 50 cm/s. However, the bifurcation area was filled with heterogeneous material, the deep and superficial femoral arteries showed no detectable flow, with diffuse sclerotic changes in the latter. Collateral circulation was present. Peripheral monophase waveform was detected only in the posterior tibial artery with a PSV of 10 cm/s. Simultaneously, sonography detected hypo-echoic material in the lumen of the external iliac, common femoral, superficial femoral, deep femoral and popliteal veins. The veins were non-compressible with no detectable flow. Due to suspicion of active intra-abdominal bleeding, a CT angiography was performed, which verified the sonographic findings and additionally revealed a secondary finding of a 55 × 22 millimeter mass in the pelvic region, indistinguishable from the external iliac vein. The final diagnosis was a tumour-altered bladder and prostate, secondary bilateral uretero-hydronephrosis and a necrotic lymph node along the course of the external iliac vein. (Fig. 3) The overall condition was concluded as simultaneous acute limb ischemia Rutherford IIb and PCD.

**Fig. 4:**
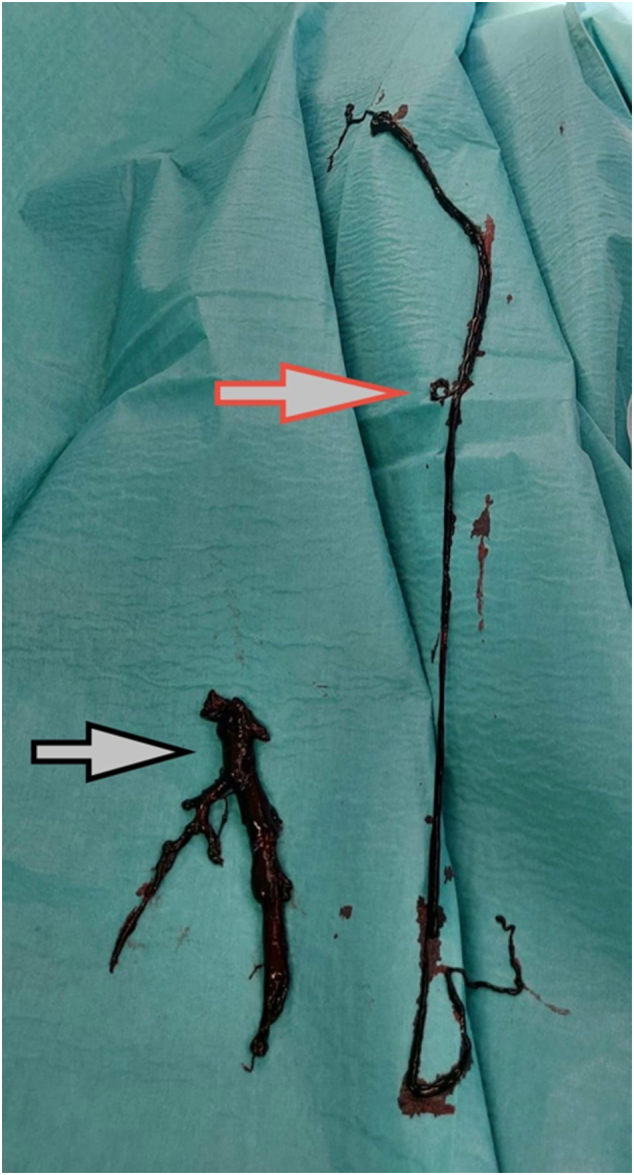
Extracted embolic material. The red arrow points to embolic material from the patient's lower limb, which was extracted without interruption using an endothelial-sparing technique. The black arrow points to embolic material retrieved from the iliac vein using Fogarty catheters. (For interpretation of the references to colour in this figure legend, the reader is referred to the web version of this article.)

Upon arrival at the emergency room, the patient was immediately administered five thousand international units of heparin alongside analgesia and oxygen therapy. The patient experienced significant and rapid progression of pain with worsening discoloration of the limb. (Fig. 1, Fig. 2). Due to Colour Duplex sonography and CT findings (acute venous and simultaneous arterial system occlusion with a newly discovered pelvic mass), clinical presentation (macroscopic haematuria, ALI Rutherford II, PCD), and laboratory findings (creatinine level 320 μmol/l, eGF-CKD-EPI 0.26 ml/s, the patient's urine output was 0.4 mL/kg/h, haemoglobin level 84 g/l), we opted for surgical intervention as a life-saving procedure. Under general anaesthesia, a single incision in the thigh provided access to both the femoral artery and vein. First, a transverse arteriotomy of the common femoral artery was performed, allowing for the extraction of thromboembolic material from the femoral artery bifurcation and the insertion of a Fogarty catheter into the deep femoral artery. Previously absent backflow was restored. The superficial femoral artery was chronically occluded. Subsequently, to minimize the damage to the venous wall during catheter manipulation, a sterile tourniquet was gradually applied from the toes toward the thigh, facilitating the evacuation of thrombotic material through a transverse venotomy made in the common femoral vein. Embolic material was extracted without interruption. (Fig. 4) A Fogarty catheter was then used to remove thrombotic material from the central segments of the venous system. Venotomy and arteriotomy sutures were applied in the standard fashion. A follow-up CT angiography was not performed due to concurrent renal failure. The patient's limb colour improved, pulse in the common femoral artery was restored, and capillary refill was observed immediately after the surgical procedure. Motor function and sensory perception of the limb returned within twelve hours postoperatively. Due to hematuria and bleeding from the pelvic tumour, postoperative anticoagulation therapy was discussed with a nephrologist. Given the high risk of potentially fatal pulmonary embolism, the patient was maintained on a reduced dose of anticoagulation therapy with regular monitoring of Anti-Xa levels. To prevent recurrent thrombus formation, hemostatic therapy was not administered. Despite this decision, hematuria did not worsen and improved following urological surgical intervention*.* Weekly follow-up with control sonography of the arterial and venous systems showed no recurrence of thrombosis. The patient remained free of recurrent deep vein thrombosis or acute limb ischemia during the one month following surgery. He was subsequently referred to the urology department for further evaluation and management. The urology department performed a left nephrostomy and transurethral resection of the bladder.

## 3 Discussion

*Phlegmasia cerulea dolens* is a rare condition that can lead to venous gangrene, limb loss, or life-threatening complications. Prompt diagnosis and treatment are crucial to prevent limb loss [8]. Current practices regarding PCD treatment remain controversial. Immediate anticoagulation is essential but often insufficient. Invasive treatments, such as catheter-directed thrombolysis and thrombectomy (including pharmacomechanical thrombectomy [PMT] and pharmacomechanical catheter-directed thrombolysis [PCDT]), are typically the primary approaches. PMT has also emerged as an effective alternative or adjunct to catheter-directed thrombolysis. Surgical thrombectomy remains a viable alternative [6,8,9]. In our case, we encountered PCD that appeared simultaneously with an acute occlusion of the deep femoral artery, leading to acute limb ischemia. The patient's overall condition was further complicated by a newly discovered pelvic tumour, acute renal failure, and acute anaemia caused by gross haematuria. We opted for open surgical intervention due to the suspected need for simultaneous arterial and venous surgery and the relative contraindication to the use of CDT due to active bleeding with signs of anaemia and the risk of using contrast agents in acute renal failure. To prevent endothelial damage during manipulation with the Fogarty catheter, we performed the venous embolectomy of the lower limb using a gradually applied compression bandage from finger to thigh, with satisfactory results. After performing the venous system embolectomy, we decided not to prolong the surgery and did not implant a preventive stent in the iliac veins due to the renal parameters and the generally poor condition of the patient on the operating table. In the postoperative period, the patient showed no signs of compartment syndrome in the affected limb and no need for fasciotomy. To prevent post-thrombotic syndrome, the patient received full anticoagulant therapy and sulodexide. In this specific patient, we hypothesize that the development of PCD was due to the compression of the iliac vessels by the pelvic mass in the presence of malignancy, which is a risk factor for the development of deep vein thrombosis. The simultaneous arterial occlusion occurred due to multiple factors. The suspected causes were previously unrecognized and untreated atherosclerotic disease of the main arterial vessels, compression of the arterial vessels as a complication of PCD, and a hypercoagulable state associated with malignancy.

## 4 Conclusion

*Phlegmasia cerulea dolens* is a rare and emergent condition requiring prompt diagnosis and treatment to prevent limb loss or life-threatening complications. Unfortunately, the literature primarily consists of isolated case reports, with a dearth of case series. Most cases are managed with endovascular techniques, and surgical thrombectomy is rarely reported. We propose that in rare instances of simultaneous acute venous and arterial thrombosis, surgical thrombectomy may be the most appropriate approach, especially given its potential for rapid execution. When feasible, endothelium-sparing thrombus removal techniques should be considered. Moreover, we believe this case highlights the necessity of training vascular surgeons in both endovascular and open-surgical techniques for venous system interventions.

The authors declare that they have no known competing financial interests or personal relationships that could have appeared to influence the work reported in this paper. We wish to confirm that there are no known conflicts of interest associated with this publication. Written consent to publish potentially identifying information, such as details or the case and photographs, was obtained from the patient. Ethics approval is not required for case reports at our institution.

## Guarantor

The Guarantor for the work is Šimon Smoter.

## Research registration number

This report is not applicable to this topic.

## CRediT authorship contribution statement

Šimon Smoter = surgical procedure performance, study concept development, original draft writing, article submission.

Roman Slyško = surgical procedure supervision, patient follow-up, scientific article correction, photography.

## Consent

Written informed consent was obtained from the patient for publication of this case report and accompanying images. A copy of the written consent is available for review by the Editor-in Chief of this journal on request.

## Ethical approval

Ethics approval is not required for case reports at our institution. University hospital of Bratislava.

## Funding

There are no funding sources.

All authors read the manuscript and approved the final manuscript.

## Declaration of competing interest

The authors declare that they have no known competing financial interests or personal relationships that could have appeared to influence the work reported in this paper.
